# A composite ^18^F-FDG PET/CT and HER2 tissue-based biomarker to predict response to neoadjuvant pertuzumab and trastuzumab in HER2-positive breast cancer (TBCRC026)

**DOI:** 10.1016/j.breast.2025.104432

**Published:** 2025-03-01

**Authors:** Maeve A. Hennessy, Ashley Cimino-Mathews, Jodi M. Carter, Jennifer M. Kachergus, Yaohua Ma, Jeffrey P. Leal, Lilja B. Solnes, Vandana G. Abramson, Lisa A. Carey, Mothaffar Rimawi, Jennifer Specht, Anna Maria Storniolo, Christos Vaklavas, Ian Krop, Eric Winer, Rita Denbow, Vincente Valero, Antonio C. Wolff, Richard L. Wahl, Chiung-Yu Huang, Vered Stearns, E. Aubrey Thompson, Roisin M. Connolly

**Affiliations:** a Cancer Research @UCC, University College Cork, Ireland; b Sidney Kimmel Comprehensive Cancer Center, Johns Hopkins School of Medicine, Baltimore, MD, USA; c Department of Laboratory Medicine and Pathology, University of Alberta, Edmonton, Canada; d Mayo Clinic Comprehensive Cancer Center, Mayo Clinic, FL, USA; e Vanderbilt University, Nashville, TN, USA; f University of North Carolina, Chapel Hill, NC, USA; g Baylor College of Medicine, Houston, TX, USA; h University of Washington, Seattle, WA, USA; i Melvin and Bren Simon Comprehensive Cancer Center, Indiana University, Indianapolis, IN, USA; j University of Alabama, Birmingham, AL, USA; k Yale Cancer Center, New Haven, CT, USA; l MD Anderson Cancer Center, Houston, TX, USA; m Washington University, St. Louis, MO, USA; n University of California, San Francisco, CA, USA

**Keywords:** ^18^F-FDG PET/CT, HER2-Biomarkers, Breast cancer, Neoadjuvant

## Abstract

**Background:**

Early metabolic change on PET/CT was predictive of response to neoadjuvant trastuzumab/pertuzumab (HP) in TBCRC026. We hypothesized that a composite biomarker incorporating PET/CT and HER2 tissue-based biomarkers could improve biomarker performance.

**Methods:**

83 patients with estrogen receptor-negative/HER2-positive breast cancer received neoadjuvant HP alone [pathologic complete response (pCR) 22 %]. PET/CT was performed at baseline and 15 days post initiation of therapy (C1D15). Promising imaging biomarkers included ≥40 % SULmax decline between baseline and C1D15, and C1D15 SULmax ≤3. Baseline tissue-based biomarkers included HER2-enriched intrinsic subtype (72 %, 46/64; NanoString), tumor HER2 protein abundance (median log2 13.5, range log2 7.1–15.9; NanoString DSP), and HER2 3+ (83 %, 64/77; immunohistochemistry). Logistic regressions were fitted to predict pCR with HER2/PET-CT biomarkers. The C statistic assessed overall prediction power. The optimal composite score cut-off was determined by maximizing Youden's index.

**Results:**

Factors most predictive for pCR in single predictor models included C1D15 SULmax (OR 0.43; p = 0.007, c = 0.77), % reduction in SULmax (OR 1.03, p = 0.006, c = 0.72) and tumor HER2 protein abundance (OR 1.75; p = 0.01, c = 0.76). The composite of C1D15 SULmax and % reduction in SULmax and their interaction term, had improved probability (c = 0.89 from c = 0.78), with high sensitivity (100 %) and negative predictive value (100 %). The addition of tumor HER2 protein did not further improve prediction power (c = 0.90).

**Conclusion:**

The HER2/PET-CT biomarker had high prediction power for pCR, however was not superior to the prediction power of PET/CT alone. Non-invasive PET/CT biomarkers may facilitate a response-guided approach to neoadjuvant therapy, allowing intensification and de-intensification of treatment, pending further evaluation.

## 1 Introduction

Strategies that optimize the treatment decision-making paradigm for individuals with human epidermal growth factor 2 (HER2)-positive early breast cancer are imperative, as we move further into an era of highly effective novel therapies and multi-agent regimens. Neoadjuvant combination cytotoxic chemotherapy with dual HER2-targeted therapy (trastuzumab and pertuzumab, HP) is the standard of care for stage II/III HER2-positive breast cancer; yielding high pathologic complete response (pCR) rates but with associated toxicity which can be serious. There is thus a clinical need for predictive biomarkers of response and resistance to these therapies so oncologists can better select patients for this approach in routine practice [1,2].

Promising tissue-based biomarkers of response to HER2-directed therapy have included the HER2-enriched molecular intrinsic subtype and HER2 mRNA. HER2-enriched tumors account for a high proportion of early stage HER2-positive breast cancers and have been associated with improved likelihood of pCR following HER2-based neoadjuvant therapy [[3], [4], [5]]. The HER2-enriched subtype shows the highest levels of HER2 mRNA and protein and appears to be the subtype with the highest activation of the epidermal growth factor receptor (EGFR)-HER2 signaling pathway, which may result in improved efficacy of HER2-targeted drugs [6]. Another interesting area of investigation is the use of functional imaging such as ^18^F-FDG PET/CT to assess response to treatment in HER2-positive breast cancer. A growing body of evidence suggests that early incorporation of serial ^18^F-FDG PET/CT in the neoadjuvant setting provides a reliable indication of response to therapy [[7], [8], [9], [10]].

The multicenter phase II TBCRC026 study investigated the correlation between early metabolic changes on ^18^F-FDG-PET/CT and pCR in patients with stage II-III estrogen receptor (ER)-negative, HER2-positive breast cancer, receiving neoadjuvant HP without chemotherapy. The primary objective was to correlate baseline and early percentage change in maximum standardized uptake value corrected for lean body mass (SULmax) on ^18^F-FDG-PET/CT with pCR to neoadjuvant dual HER2-directed therapy. The study incorporated serial ^18^F-FDG-PET-CT imaging [baseline and 15 days after HP initiation (C1D15)] as well as tumor biopsy collection. The primary study results found that participants not obtaining a 40 % reduction in SULmax by C1D15 were unlikely to obtain pCR, with a high negative predictive value (NPV) of 91 % [9]. A secondary analysis of the TBCRC026 trial also demonstrated a potential association between SULmax parameters on ^18^F-FDG-PET/CT and survival outcomes. Notably, achieving a C1D15 SULmax ≤3 was associated with improved recurrence-free survival (HR 0.36; p = 0.06) and significantly improved overall survival (HR 0.14; p = 0.03) [11].

While these tissue- and imaging-based biomarkers have demonstrated promise in terms of their associations with treatment response in a number of studies, further validation is required before clinical practice can be influenced. At present, we continue to rely on conventional clinico-pathologic factors such as hormone receptor status, HER2 expression or amplification and pCR to guide treatment decision-making and prognostication in daily practice. It has become clear that the heterogenous nature of HER2-positive breast cancer may render the application of a single predictive biomarker challenging, and this has led to the evaluation of composite biomarkers [6,12,13].

We aimed to expand on the results of the TBCRC026 trial by combining promising HER2 tissue-based biomarkers with ^18^F-FDG PET/CT parameters in a composite biomarker analysis. The ^18^F-FDG PET/CT imaging biomarker performed extremely well in TBCRC026, with a lack of decline in SULmax at C1D15 predictive for those unlikely to achieve pCR with HP alone [9]. We thus hypothesized that a composite biomarker incorporating baseline HER2 tissue-based biomarkers in addition to ^18^F-FDG PET/CT could further improve biomarker performance in patients with early-stage HER2-positive breast cancer undergoing neoadjuvant therapy with HP alone.

## 2 Methods

### 2.1 Clinical trial design

Study design, eligibility criteria and primary endpoint results have previously been reported for the neoadjuvant TBCRC026 trial (NCT01937117) [9]. Briefly, patients with stage II-III ER-negative/HER2-positive breast cancer received 4 cycles of neoadjuvant HER2-directed therapy alone with HP (Supplementary Fig. 1). ^18^F-FDG PET/CT was performed at baseline, and C1D15, prior to research tumor biopsies [9]. The primary endpoint was to correlate early metabolic change on ^18^F-FDG PET/CT between baseline and C1D15 with pCR, following 4 cycles of HP. pCR was defined as no viable invasive cancer in the breast and axilla in the surgical specimen (local pathology review), following HP without chemotherapy [14]. Patients who had residual disease following neoadjuvant HP or clinical progression on HP were classified as non-pCR.

Baseline research tumor biopsies were collected prior to initiating HP and at C1D15, and tumor tissue was obtained from the surgical specimen at the time of surgery from those with residual disease. Only baseline samples were evaluated for the purposes of this analysis. All patients signed a written informed consent that was approved by the institutional review boards of the participating institutions.

### 2.2 ^18^F-FDG PET/CT acquisition and image analysis

^18^F-FDG PET/CT facilities required approval by the study team and study imaging manuals were provided to ensure consistency in methodology, in addition to review of representative clinical scans and phantom images [9]. ^18^F-FDG PET/CT was carried out at baseline and C1D15 with a 3-day window permitted per protocol. A combined ^18^F-FDG PET/CT scan was obtained from midskull to midfemur, following a 60-min uptake phase after administration of intravenous FDG injection. Digital transmission of scans facilitated blinded central review and quantitation [9]. All imaging procedures were conducted in conformance with the Uniform Protocols for Imaging in Clinical Trials ^18^F-FDG PET/CT and Radiological Society of North America Quantitative Imaging Biomarkers Alliance profiles [15,16]. Measurements were acquired by placing a spherical volume of interest over the target primary breast cancer tissue and recording SULmax, with avoidance of normal adjacent tissue. Regarding patient preparation, to ensure consistency, all patients were instructed to fast for a minimum of 4 h prior to imaging. Patients with diabetes required a consult with a Nuclear Medicine Physician prior to the 18F-FDG PET/CT to coordinate procedures for scheduling and imaging. If there were any imaging concerns that the patient may not be suitable for quantitative ^18^F-FDG PET/CT, discussion with the local and central radiologists was required to confirm eligibility for the trial. Further detailed information is available in the Supplementary Material (see protocol, Appendix D) of the primary TBCRC026 publication [9].

### 2.3 ^18^F-FDG PET/CT biomarkers

^18^F-FDG PET/CT imaging parameters included in this composite biomarker analysis were predefined based on the following primary results from TBCRC026. Results of both univariable and multivariable logistic regression revealed that median percent reduction in SULmax at C1D15 differed significantly between those with pCR and without pCR post HP (64 % versus 42 %; p = 0.004; Table 1). In a receiver operating characteristic (ROC) analysis, SULmax decline ≥40 % by C1D15, (ΔSULmaxD15 ≥ 40 %) had high sensitivity (83 %) and NPV (91 %) for predicting lack of pCR, and this threshold was considered clinically optimal [9]. A significant difference was also observed in the median C1D15 SULmax value between those who achieved pCR and those who did not (1.6 versus 3.6; p < 0.001; Table 1). An exploratory cut off of ≤3 versus >3 for C1D15 SULmax was tested and a higher proportion of patients who obtained pCR had a C1D15 SULmax ≤3 compared to those who did not (100 % v 45 %; P < 0.001). Compared to other cut points examined, this cut off of ‘3’ had a high NPV (100 %) for predicting pCR [9]. Based on the above findings from the primary analysis, the following ^18^F-FDG PET/CT parameters were assessed in the current analysis: C1D15 SULmax (mean, median, range), C1D15 SULmax (≤3 versus >3), % reduction in SULmax (mean, median, range) and % reduction in SULmax (<40 % versus ≥40 %).

**Table 1:** Tissue-Based and PET/CT Biomarker Characteristics by pCR status.

	No pCR (N = 65)	pCR (N = 18)	Total (N = 83)	P value
**Tissue-Based Biomarkers**				
*Intrinsic Subtype*				0.32
Basal + Luminal A	16 (32 %)	2 (14 %)	18 (28 %)	
HER2 Enriched	34 (68 %)	12 (86 %)	46 (72 %)	
*HER2 Expression (IHC)*				0.44
0/1+/2+	12 (19 %)	1 (7 %)	13 (17 %)	
3+	50 (81 %)	14 (93 %)	64 (83 %)	
Log*2 HER2 protein tumor (DSP)*				0.002
Mean (SD)	12.3 (2.4)	14.2 (1.4)	12.7 (2.3)	
Median (Range)	13.2 (7.1–15.8)	14.5 (9.8–15.9)	13.5 (7.1–15.9)	
**PET/CT Biomarkers**				
*C1D15 SULmax*				<0.001
Mean (SD)	4.6 (3.9)	1.7 (0.6)	3.9 (3.7)	
Median (Range)	3.6 (0.6–15.9)	1.6 (0.9–2.8)	2.4 (0.6–15.9)	
*% Reduction in SULmax*				0.004
Mean (SD)	38.3 (32.2)	62.9 (21.3)	43.6 (31.7)	
Median (Range)	41.8 (−34.9–91.9)	63.8 (25.1–91.5)	52.5 (−34.9–91.9)	
*D15 SULmax*				<0.001
>3	36 (55 %)	0 (0 %)	36 (43 %)	
≤3	29 (45 %)	18 (100 %)	47 (57 %)	
*% Reduction in SULmax*				0.03
<40 %	31 (48 %)	3 (17 %)	34 (41 %)	
≥40 %	34 (52 %)	15 (83 %)	49 (59 %)	

Abbreviations: SULmax, standardized uptake by lean body mass; pCR, pathologic complete response; C1D15, Cycle 1 Day 15; IHC, immunohistochemistry; DSP, digital spatial profiling; P-values were obtained using Fisher's exact test for comparing binary variables and Mann–Whitney *U* test for comparing continuous variables.

### 2.4 HER2 tissue based biomarkers

Baseline tumor specimens were analyzed for individual biomarkers based on tissue availability. For the purposes of this composite biomarker analysis we predefined what were felt to represent the most promising baseline HER2 tissue-based biomarkers based on the current literature. This included HER2 overexpression (score 3+) assessed by central immunohistochemistry (IHC) according to American Society of Clinical Oncology/College of American Pathologists guidelines [17]; high HER2 protein abundance (tumor and stroma) evaluated using NanoString Digital Spatial Platform; and HER2-enriched intrinsic subtype, assessed using the NanoString BC360 platform.

### 2.5 Statistical analysis

The exploratory HER2/PET-CT biomarkers were compared between patients with and without pCR using Fisher's exact tests and Wilcoxon rank-sum tests, as appropriate. The associations between the exploratory composite HER2/PET-CT biomarker and pCR were evaluated using logistic regressions, where p values were obtained using Wald-type tests. The overall discriminatory power of the predictive models was evaluated using the c-statistic, that is, the area under the ROC curve. The c-statistic measures the concordance between model-based risk estimates and observed binary outcome status. A larger c-statistic signifies better discriminatory performance, with 0.5 indicating no discriminative ability (equivalent to random guess) and 1.0 representing perfect discrimination. Sensitivity and specificity were evaluated at the optimal cut-off point determined by maximizing Youden's index. In all analyses, two-sided p values less than 0.05 were considered statistically significant.

## 3 Results

### 3.1 Patient Demographics

Clinico-pathologic characteristics from TBCRC026 were reported previously and are summarized in Supplementary Table 1. Enrollment of 88 women took place from January 2014–August 2017, with 83 evaluable for this composite biomarker analysis. All four cycles of neoadjuvant HP were completed by 85 % of patients with additional non-study pre-operative therapy administered to 25 patients (28 %), and these patients were classified as not obtaining pCR. The pCR rate was 22 % (18/83) following four cycles of HP without chemotherapy.

### 3.2 ^18^F-FDG PET/CT biomarkers

To summarize the previously reported results for the ^18^F-FDG PET/CT parameters, the % reduction in SULmax was significantly associated with pCR (p = 0.004), with a median of 52.5 % (range −34.0-91.9). ΔSULmaxD15 ≥ 40 % was achieved in 59 % (49/83) of patients and was associated with pCR (p = 0.03). The median C1D15 SULmax value differed significantly between those achieving pCR and those who did not (p < 0.001), and a C1D15 SULMax ≤3 was achieved in 57 % (47/83) of patients and was significantly associated with pCR, p = 0.001 (Table 1) [9].

### 3.3 HER2-tissue based biomarkers

In line with emerging biomarkers of interest, baseline HER2 tissue-based biomarkers examined intrinsic subtype, HER2 expression by IHC and HER2 tumor protein abundance by digital spatial profiling with a summary of results presented in Supplementary Table 1. The HER2-enriched intrinsic subtype was present in 72 % of patients (46/64 evaluable). Central evaluation of HER2 by IHC revealed high expression of HER2 (score 3+) in 83 % of patients (64/77 evaluable). HER2 protein abundance by digital spatial profiling was analyzed in 71/83 samples, with median log 2 HER tumor protein abundance of 13.5 (range 7.1–15.9) and median log2 HER2 stroma protein abundance of 10.2 (range 5.1–13.9). HER2 tumor protein abundance differed significantly between those who obtained pCR and those who did not, p = 0.002 (Table 1).

### 3.4 Composite biomarker performance

**Fig. 1:**
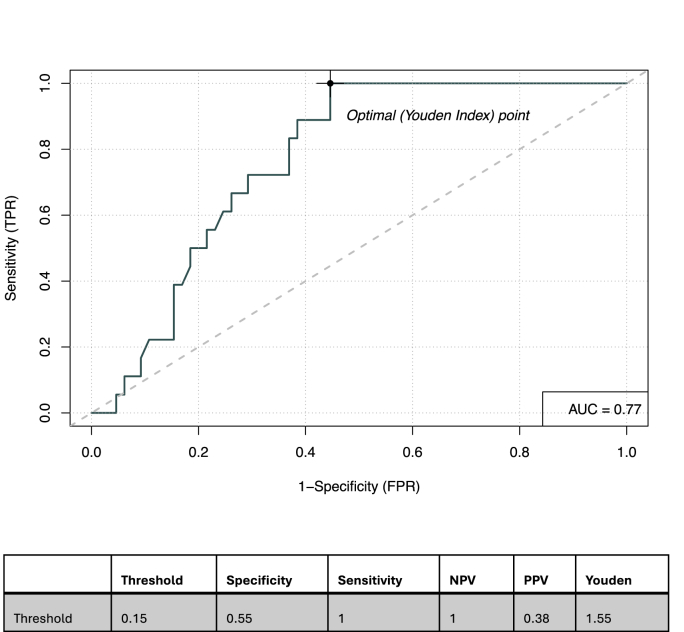
Receiver Operating Characteristic curve for C1D15 SULmax. Abbreviations: AUC: Area Under the Curve, NPV: Negative Predictive Value, PPV: Positive Predicitive Value.

In the single variable prediction model, the ^18^F-FDG PET/CT parameters most predictive for pCR included C1D15 SULmax (OR 0.43; p = 0.007, c = 0.77) and percent reduction in SULmax (OR 1.03, p = 0.006, c = 0.72) (Table 2). Regarding the HER2-tissue based biomarkers, HER2 tumor protein abundance was the most promising for prediction of pCR, (OR 1.75; p = 0.01, c = 0.76), while HER2-enriched and HER2 3+ expression had lower concordance (c = 0.59, p = 0.2 and c = 0.56, p = 0.3, respectively) (Table 2). The ROC analysis, with C1D15 SULmax as a single predictor, had a specificity of 55 %, sensitivity of 100 %, NPV 100 % and positive predictive value (PPV) 38 % and yielded a c statistic of 0.77 (Fig. 1).

**Table 2:** Univariate logistic regression analysis of pCR and prediction performance of HER2 and PET/CT Biomarkers.

	OR	95 % CI	P value	Concordance
**HER2-Enriched**	2.82	(0.67, 19.5)	0.21	0.59
**HER2 3+ (IHC)**	3.36	(0.58, 63.8)	0.26	0.56
**Log2 HER2 protein tumor**	1.75	(1.20, 2.97)	0.01	0.76
**C1D15 SULmax**	0.43	(0.21, 0.71)	0.007	0.77
**% Reduction in SULmax**	1.03	(1.01, 1.06)	0.006	0.72

Abbreviations: IHC, immunohistochemistry; SULmax, standardized uptake by lean body mass; C1D15, Cycle 1 Day 15; OR, odds ratio; CI, 95 % confidence interval of the odds ratio; Concordance is measured by C-statistic.

**Fig. 2:**
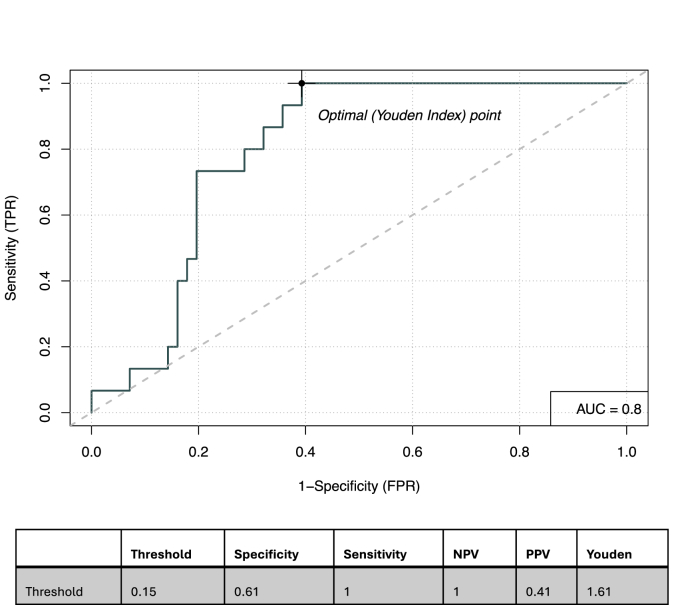
Receiver Operating Characteristic curve for the composite of D15SULmax and Log 2 HER2 tumor protein abundance. Abbreviations: AUC: Area Under the Curve, NPV: Negative Predictive Value, PPV: Positive Predicitive Value.

Investigating composite biomarkers that included C1D15 SULmax as a component did not appear to meaningfully improve prediction power (Supplementary Table 2). For example, the composite biomarker of C1D15 SULmax and log2 HER2 protein tumor had a specificity of 61 %, sensitivity 100 %, NPV 100 % and PPV 41 % (Fig. 2). Similarly, the composite of C1D15 SULmax and percent reduction in SULmax had a specificity of 60 %, sensitivity 100 %, NPV 100 % and PPV 41 %, c = 0.78 (Fig. 3A).

**Fig. 3A:**
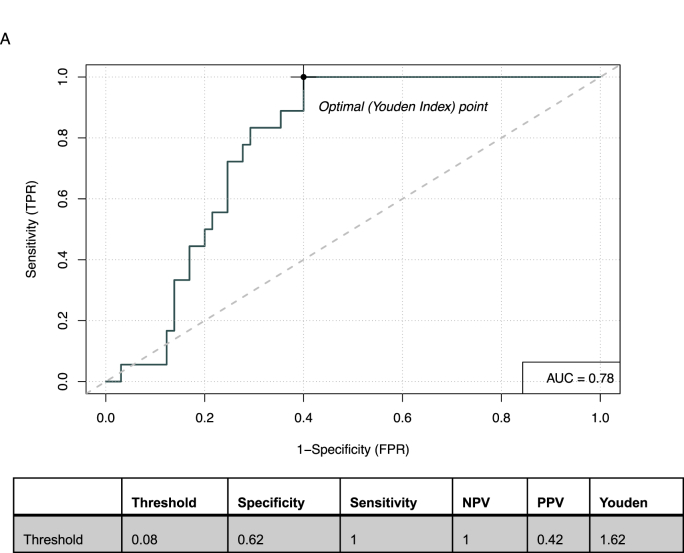
Receiver Operating Characteristic curve for the composite of D15 SULmax and % reduction in SULmax. Abbreviations: AUC: Area Under the Curve, NPV: Negative Predictive Value, PPV: Positive Predicitive Value.

However, including the interaction term between C1D15 SULmax and percent reduction in SULmax in the prediction model improved the concordance probability, increasing from c = 0.78 to c = 0.89 (Fig. 3B). This resulted in high sensitivity (100 %) and NPV (100 %) (Table 3A). The inclusion of the interaction term allows the effect of C1D15 SULmax on predicting pCR to vary across different levels of % reduction in SULmax (or vice versa), and Table 3A suggests that there is a synergistic effect between these 2 variables and pCR. This composite endpoint provided a similar discrimination power when compared to the model with the three biomarkers: D15 SULmax, % reduction in SULmax, their interaction term, and tumor HER2 protein abundance (c = 0.90), i.e. the addition of HER2 tumor protein abundance, which was the most promising HER2 biomarker in the single prediction model, did not further improve prediction power (Table 3B).

**Fig. 3B:**
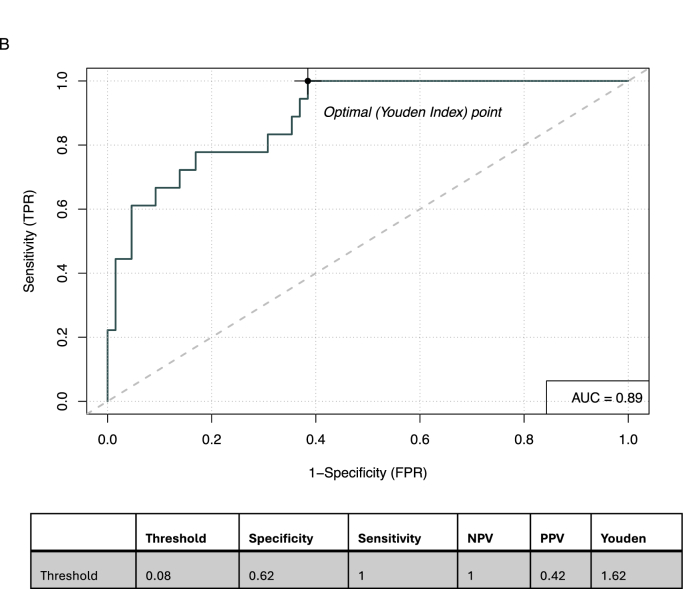
Receiver Operating Characteristic curve for the composite of D15 SULmax and % reduction in SULmax including interaction term. Abbreviations: AUC: Area Under the Curve, NPV: Negative Predictive Value, PPV: Positive Predicitive Value.

**Table 3A:** Multivariate logistic regression analysis correlating pCR with C1D15 SULmax, percent reduction in SULmax, and their interaction term.

	OR	95 % CI	P value	Concordance
				0.89
**C1D15 SULmax**	0.0005	(0.00, 0.05)	0.01	
**% Reduction in SULmax**	0.84	(0.71, 0.95)	0.02	
**Interaction between C1D15 SULmax and % Reduction in SULmax**	1.12	(1.04, 1.25)	0.01	

Abbreviations: C1D15, Cycle 1 Day 15; SULmax, standardized uptake by lean body mass; OR, odds ratio; CI, 95 % confidence interval of the odds ratio; Concordance is measured by C-statistic.

**Table 3B:** Multivariate logistic regression analysis correlating pCR with C1D15 SULmax, % reduction in SULmax, their interaction term, and HER2 protein abundance.

	OR	95 % CI	P value	Concordance
				0.90
**C1D15 SULmax**	0.0002	(0.00, 0.05)	0.02	
**% Reduction in SULmax**	0.81	(0.65, 0.94)	0.02	
**Interaction between C1D15 SULmax and % Reduction in SULmax**	1.14	(1.04, 1.30)	0.02	
**Log2 HER2 protein tumor**	1.35	(0.76, 2.73)	0.34	

Abbreviations: C1D15, Cycle 1 Day 15; SULmax, standardized uptake by lean body mass; OR, odds ratio; CI, confidence interval.

## 4 Discussion

The TBCRC026 primary imaging trial investigated the correlation between early metabolic changes in SULmax on ^18^F-FDG PET/CT and pCR to HER2-directed therapy alone (HP), without chemotherapy. We previously showed that a lack of decline in SULmax at C1D15 best predicts those unlikely to achieve pCR with HP alone, with a high NPV of 91 % [9,11]. In a secondary survival analysis, we demonstrated that the D15 SULmax threshold of 3 was correlated with survival; with those achieving a C1D15 SULmax ≤3 having a significantly improved OS (p = 0.03) [11]. In this composite analysis, combining the C1D15 SULmax value and the interim percent reduction in SULmax had a high sensitivity (100 %) and NPV (100 %) for pCR. Contrary to our hypothesis that a composite biomarker might improve biomarker performance, the addition of a HER2 biomarker (high HER2 protein abundance) to this model had high prediction power (c = 0.9) however did not significantly strengthen the performance of the ^18^F-FDG PET/CT biomarker (c = 0.89).

Several other studies have explored the use of ^18^F-FDG PET/CT as an early imaging biomarker of response to HER-directed therapy and concluded that it may allow for early prediction of response, however these studies are heterogeneous in design and this approach is not yet standard of care [10,[18], [19], [20]]. The recent PHERGain study explored a response-adapted approach in the treatment of early-stage HER2-positive breast cancer, with patients randomized to either neoadjuvant chemotherapy plus HP or HP alone. Almost 80 % of patients that received HER2-directed therapy alone achieved a metabolic response on ^18^F-FDG PET/CT (defined as an SUV decline of ≥40 % from baseline after 2 cycles of treatment) with an excellent 3 year invasive disease-free survival of 95 % [10]. Updated results also demonstrated that in 37 % of those who responded on ^18^F-FDG PET/CT, a pCR was achieved, and thus there was a subgroup who never received chemotherapy, yet had a 3 year invasive disease-free survival of 98.8 % [19]. The value of an SUV threshold on interim ^18^F-FDG PET/CT, rather than the decline in SUV as a predictive marker of response has also been demonstrated by others investigating HER2-positive breast cancer. For example, Groheux et al. showed that the absolute residual SUVmax value after two cycles of chemotherapy was most predictive for pCR when compared to baseline SUVmax and change in SUVmax, and that the risk of not achieving pCR was 92 % in patients with residual uptake >3 at the second scan (p = 0.0001) [21]. Similarly, Humbert et al. demonstrated that a very low SUVmax (<2.1) after once cycle of systemic therapy was the main predictor of pCR (p = 0.004) [22]. An important distinction is that the patients in these earlier trials received chemotherapy in combination with HER2-directed therapy, as opposed to HER2-directed therapy alone as explored in TBCRC026. The ongoing ECOG-ACRIN EA1211/DIRECT trial aims to validate ^18^F-FDG PET/CT as a neoadjuvant imaging integral biomarker in patients treated with standard HER2-directed regimens, using the threshold of 40 % decline in SULmax at C1D15 identified in the TBCRC026 study [23]. If the trial meets its objectives, future clinical utility studies will examine the ability of the PET/CT biomarker to adapt therapy for patients with early-stage HER2-positive breast cancer. It is envisaged that PET/CT may help to quickly identify patients (e.g. those not achieving a 40 % decline in SULmax by C1D15) who need therapy escalation, alternative regimens or clinical trials, versus those who are likely to achieve a pCR with less toxic regimens. We eagerly await the results from EA1211/DIRECT which could potentially lead to a shift in clinical practice by enabling a ‘Response Guided Treatment Strategy’ using ^18^F-FDG PET/CT as a standalone tool to tailor therapy.

In the interim, we have explored how to further improve the biomarker performance of PET/CT by incorporating promising HER2 tissue-based biomarkers into this composite biomarker analysis. The HER2 biomarkers chosen were predefined in the primary protocol based on existing clinically relevant biomarkers, such as HER2 expression on IHC, and review of the literature at the time of protocol development. Those that appeared most promising on univariate analysis were then incorporated into the multivariate analysis. Given the significant heterogeneity among HER2-positive breast cancers, relying on a single predictive biomarker is likely to be challenging and instead we anticipate that future prognostic models will integrate multiple variables to guide treatment decision-making [24]. Our approach combines biological data from HER2 tissue-based biomarkers, which reflects the tumor's molecular characteristics and potential for response to HER2-targeted therapy, with functional information from PET imaging which assesses metabolic activity and monitors dynamic response to therapy in real time, with the aim of creating a personalized predictive tool. Efforts examining combination HER2 tissue-based biomarkers have recently yielded interesting and hypothesis-generating results. In an analysis of tumors from five clinical trials of early HER2-positive breast cancer treated with neoadjuvant HER2-directed therapy alone, an RNA-based assay combining both ERBB2 and the HER2-enriched intrinsic subtype allowed better identification of high anti-HER2 sensitivity compared to when either were used in isolation [6]. A combinatorial biomarker of HER2 amplification plus markers of the phosphoinositide 3-kinases (PI3K) pathway activation and pCR was also examined in an analysis of TBCRC006 trial, which investigated neoadjuvant lapatinib/trastuzumab [12,25]. A high level of HER2 amplification combined with wild-type PI3K pathway status was associated with pCR (p = 0.0031) and may indicate increased sensitivity to HER2-targeted therapies without chemotherapy [12]. While composite tissue-based biomarkers have been explored by investigators in the HER2-positive setting, such as in the above studies, the incorporation of imaging and tissue-based biomarkers in the assessment of response to HER2-directed therapy without chemotherapy in early breast cancer is relatively unique. We chose to focus on the HER2-enriched subtype, high HER2 amplification and high levels of HER2 protein abundance for this composite analysis, based on promise previously demonstrated in other trials as well as based on available correlative data from this study [4,12,26,27]. For example, tumors that had a high level of HER2 protein abundance in our analysis were more likely to achieve pCR (p = 0.002). However, although combining this with the C1D15 SULmax value and % reduction in SULmax on ^18^F-FDG PET/CT did yield a high C value of 0.9, it did not significantly improve upon the performance of the imaging biomarkers. It is likely that this is due to the fact that the ^18^F-FDG PET/CT performed exceptionally well in terms of predicting response to therapy.

Potential limitations of our study include a relatively small patient cohort. In addition, not all of the tissue samples were evaluable for all analyses, which may account for the fact that the combination biomarker did not significantly improve on the performance of the imaging biomarker alone. The attrition in samples is in many cases an unavoidable limitation of tissue-based biomarker analysis, and renders the non-invasive ^18^F-FDG PET/CT biomarker attractive. However, access to ^18^F-FDG PET/CT imaging and associated costs are important considerations, particularly in resource-limited settings. This was an exploratory analysis and as such our findings should be considered as hypothesis-generating. Note that the concordance analysis was not cross-validated, hence it may over-estimate the performance of the prediction model. Future trials incorporating larger, diverse cohorts and cross-validation of predictive models in larger datasets to ensure robustness are warranted. Despite these limitations, our study highlights the novel concept of combining imaging and tissue biomarkers and is one of the first studies to our knowledge to have examined this in the neoadjuvant HER2-positive setting. Going forward, the potential of combining different predictive biomarkers to identify HER2-positive patients who may benefit from HER2-targeted therapies, without the need for chemotherapy or with less toxic chemotherapy regimens, is likely to be a key area of research focus.

## 5 Conclusion

The composite HER2/PET-CT biomarker comprising D15 SULmax, % reduction in SULmax and tumor HER2 protein abundance had high prediction power for pCR, however was not significantly superior to the prediction power of ^18^F-FDG PET/CT parameters and their interaction term alone. These data suggest that clinical trials informed by early ^18^F-FDG PET/CT biomarkers warrant further evaluation to personalize breast cancer care. Ideally, with thoughtfully designed studies and global collaboration amongst the breast oncology community we will continue to progress validation and clinical utility studies in order to bring the most promising biomarkers into the clinic, enabling optimization of therapy.

## CRediT authorship contribution statement

**Maeve A. Hennessy:** Writing – review & editing, Writing – original draft, Visualization, Methodology, Formal analysis, Conceptualization. **Ashley Cimino-Mathews:** Writing – review & editing, Investigation. **Jodi M. Carter:** Writing – review & editing, Investigation, Formal analysis, Data curation. **Jennifer M. Kachergus:** Writing – review & editing, Validation, Resources, Methodology, Investigation, Formal analysis, Data curation. **Yaohua Ma:** Writing – review & editing, Formal analysis, Data curation. **Jeffrey P. Leal:** Writing – review & editing, Software, Data curation. **Lilja B. Solnes:** Writing – review & editing, Visualization, Investigation, Conceptualization. **Vandana G. Abramson:** Writing – review & editing, Resources, Investigation. **Lisa A. Carey:** Writing – review & editing, Conceptualization. **Mothaffar Rimawi:** Writing – review & editing, Visualization, Resources, Investigation. **Jennifer Specht:** Writing – review & editing, Resources, Data curation. **Anna Maria Storniolo:** Writing – review & editing, Resources. **Christos Vaklavas:** Writing – review & editing, Resources, Investigation. **Ian Krop:** Writing – review & editing, Supervision, Conceptualization. **Eric Winer:** Writing – review & editing, Visualization, Validation, Supervision, Software, Resources, Project administration, Methodology, Investigation, Funding acquisition, Formal analysis, Data curation, Conceptualization. **Rita Denbow:** Writing – review & editing, Project administration, Data curation. **Vincente Valero:** Writing – review & editing, Resources, Investigation. **Antonio C. Wolff:** Writing – review & editing, Resources, Investigation, Funding acquisition, Formal analysis, Conceptualization. **Richard L. Wahl:** Methodology, Formal analysis, Conceptualization. **Chiung-Yu Huang:** Writing – review & editing, Formal analysis. **Vered Stearns:** Writing – review & editing, Resources, Methodology, Investigation, Conceptualization. **E. Aubrey Thompson:** Writing – review & editing, Supervision, Project administration, Methodology, Funding acquisition, Formal analysis, Conceptualization. **Roisin M. Connolly:** Writing – review & editing, Visualization, Supervision, Resources, Project administration, Methodology, Formal analysis, Conceptualization.

## Disclosures

**Maeve Hennessy:** Support for meetings/travel: Roche, MSD, AstraZeneca.

**Vandana Abramson:** Consulting Fees: Guardant Health, AstraZeneca.

**Mothaffar Rimawi:** Grants: Pfizer, Consulting Fees: Macrogenics, SeaGen, Novartis, AstraZeneca, Pfizer. Patents - Co-inventor PCT/US21/70543 (Methods for breast cancer treatment and prediction of therapeutic response) pending to Baylor College of Medicine.

**Jodi Carter:** Advisory board: Agilent, AstraZeneca, Roche.

**Christos Vaklavas:** Grants to institution: Pfizer, SeaGen, H3 Biomedicine/Eisai, AstraZeneca, CytomX, Daiichi Sankyo; Consulting Fees: Guidepoint, Novartis, SeaGen, Daiichi Sankyo, AstraZeneca, Gilead, Genentech; Honoraria: Gilead, AstraZeneca, Patents - Breast Cancer Diagnostic. Patent No. 63/133,678 (pending), Leadership role - Society of Utah Medical Oncologists (Board Member, unpaid), other: Flatiron (employment) Wife, Genentech (Think Tank, unpaid)

**Ian Krop:** Grants (institution): Pfizer, Macrogenics, Genentech/Roche; consulting fees: AstraZeneca, Daiichi Sankyo, Genentech/Roche, SeaGen; Advisory Board: SeaGen, Novartis, Merck.

**Richard Wahl:** Consulting/Advisory board: Clarity Pharmaceuticals, Voximetry, MTI, Molecular Targeting Technologies, Abdera, Seno; Stock: Clarity Pharmaceuticals, Honoraria: Siemens, Radiopharma; Research funding: Bayer, Viewpoint (Perspective), Fusion Pharma, Rayze, Fusion, Siemens Healthineers, ITM, White Rabbit AI, Actinium; Travel: Sidra Health Qatar, Perspective, Siemens, other: Past President SNMMI (uncompensated)

**Vered Stearns:** Research grants (institution): Abbvie, Biocept, Novartis, Pfizer, Puma Biotechnology, QUE Oncology. DSMB: Chair, AstraZeneca; Chair, ASCO, TAPUR study Non-financial support: Foundation Medicine Study Assays.

**Roisin Connolly:** Research Funding: MSD, Pfizer, Daichii Sankyo, AstraZeneca (institution). Support for meetings/travel: Gilead, Novartis, Roche. Advisory Board - Non-Financial: Seagen (Chair), Financial: AstraZenca/Daichii Sankyo, Gilead. Steering Committee member: AstraZeneca/Daichii (financial) and Develop Med-UCD, ACRI, Decrescendo (Non-financial)

No other conflicts of interest reported.

## Research support

TBCRC and its foundation partners (The Breast Cancer Research Foundation and Susan G. Komen for the Cure), ASCO CCF CDA 2013, Genentech (ML28190), SKCCC Core Grant (P30-CA006973), AVONCenter of Excellence (01-2017-007), NCI Quantitative Imaging 10.13039/100031212Network (QIN) contract (5U01CA140204), 10.13039/100001006Breast Cancer Research Foundation (grant 21-161)
