# Supplementary material for: A composite 18F-FDG PET/CT and HER2 tissue-based biomarker to predict response to neoadjuvant pertuzumab and trastuzumab in HER2-positive breast cancer (TBCRC026)
Source: Breast. 2025 Mar 1;81:104432. doi: 10.1016/j.breast.2025.104432 (PMC11928837; doi:10.1016/j.breast.2025.104432)
Supplement: Multimedia component 1 [file mmc1.docx]

Abbreviations: AUC: Area Under the Curve, NPV: Negative Predictive Value, PPV: Positive Predicitive Value

| Characteristic | *n* = 83 (%) |
| --- | --- |
| Age (years) |  |
| Median, Range | 58 (29-82) |
| Tumor Size (cm) |  |
| Median, Range | 3.9 (2-15) |
| Baseline Clinical Stage |  |
| II | 71 (86) |
| III | 12 (14) |
| Tumor Grade |  |
| 2 | 20 (24) |
| 3 | 63 (76) |
| HER2 IHC | **N = 77** |
| 1+ | 4 (5%) |
| 2+ | 9 (12%) |
| 3+ | 64 (83%) |
| Intrinsic Subtype | **N = 64** |
| Basal | 16 (25%) |
| HER2 Enriched | 46 (72%) |
| Luminal A | 2 (3%) |
| log2 HER2 protein abundance (stroma) | **N = 71** |
| Median (Range) | 10.2 (5.1-13.9) |
| log2 HER2 protein abundance (tumor) |  |
| Median (Range) | 13.5 (7.1-15.9) |

**Supplementary Table 1: Summary of clinicopathologic characteristics (evaluable population)**

Abbreviations: IHC, immunohistochemistry; HER2, human epidermal growth factor 2

|  | OR | 95% CI | P  value | Concordance |
| --- | --- | --- | --- | --- |
| HER2 Enriched | 1.71 | (0.32, 13.6) | 0.56 | 0.73 |
| C1D15 SULmax | 0.5 | (0.23, 0.82) | 0.03 |  |
| HER2 3+ | 0.53 | (0.04, 13.4) | 0.65 | 0.77 |
| C1D15 SULmax | 0.44 | (0.2, 0.74) | 0.01 |  |
| Log2 HER2 protein abundance (tumor) | 1.45 | (0.9, 2.7) | 0.17 | 0.80 |
| C1D15 SULmax | 0.49 | (0.23, 0.83) | 0.03 |  |
| % Reduction in SULmax | 1.01 | (0.99, 1.04) | 0.5 | 0.78 |
| C1D15 SULmax | 0.47 | (0.22, 0.82) | 0.02 |  |

**Supplementary Table 2: Composite biomarkers including D15 SULmax as a component**

Abbreviations: HER2, human epidermal growth factor 2; C1D15, cycle 1 day 15; SULmax, standardized uptake by lean body mass; OR, odds ratio; CI, 95% confidence interval of the odds ratio; Concordance is measured by C-statistic.

**Supplementary Figure 1: TBCRC026 Study Schema**

Abbreviations: T, tumor size; N, nodes; HER2, human epidermal growth factor 2; ER, estrogen receptor; PR, progesterone receptor; PET, positron emission tomography; C1D15, cycle 1 day 15; q21d, every 21 days
